# New Advances in Gastroenterology: The Crucial Role of Molecular Medicine

**DOI:** 10.3390/ijms241914907

**Published:** 2023-10-05

**Authors:** Marcello Candelli, Francesco Franceschi

**Affiliations:** Department of Emergency, Anesthesiological and Reanimation Sciences, Fondazione Policlinico Universitario A. Gemelli–IRCCS, 000135 Rome, Italy; francesco.franceschi@policlinicogemelli.it

The significant progress we have recently observed in the field of gastroenterology, both in the understanding of pathophysiological mechanisms and in the diagnosis and treatment of diseases, is closely related to the improvement and discovery of new biomolecular techniques. Metagenomics and metatranscriptomics have enabled researchers to identify and analyze the multitude of microbial species present in the gut and their functional genes [1]. These advances have led to a better understanding of how specific microbial species influence gastrointestinal health and have opened the potential for developing targeted interventions to modulate the gut microbiota. The gut microbiota plays a critical role in several physiological functions, including nutrient metabolism, immune regulation, and enteric nervous system development. Studies have shown that the gut microbiome can communicate with the brain through the vagus nerve and the production of neurotransmitters such as serotonin [2]. An imbalance in the gut microbiota (dysbiosis) has been associated with gastrointestinal disorders such as inflammatory bowel disease and irritable bowel syndrome [3]. In addition, the gut–brain axis has been associated with mental illnesses such as anxiety and depression [2]. In this context, the study of H. pylori can be considered as a paradigm for bacterium–host interactions, which, on the one hand, may lead to evaluating the beneficial effects of this interaction and, on the other hand, lead to a better understanding of the pathological mechanisms that may lead to inflammatory diseases or neoplastic developments [4]. Other techniques such as CRISPR-Cas9 have proven to be powerful tools to study genetic factors contributing to gastrointestinal diseases. By introducing specific genetic alterations into animal models, researchers can mimic the genetic changes observed in human patients and study their effects on gastrointestinal development and function. Inflammatory bowel disease (IBD) is one area where CRISPR-Cas9 has made an important contribution. Researchers have used this technology to create mouse models with genetic mutations associated with IBD, gaining valuable insights into the molecular pathways underlying disease development [5]. In addition, CRISPR-Cas9 has enabled researchers to perform functional studies on the candidate genes involved in gastrointestinal diseases such as colorectal cancer [6]. By precisely editing the genes of interest, researchers can determine the role of the genes in maintaining intestinal barrier integrity, regulating immune responses, and modulating small bowel motility. Other molecular biology techniques, such as genome-wide DNA methylation profiling and chromatin immunoprecipitation sequencing (ChIP-seq), have enabled researchers to detect epigenetic changes throughout the genome. These advances have uncovered the epigenetic landscape of gastrointestinal cancer and led to the discovery of potential biomarkers for early detection and the development of epigenetic-targeted therapies. Epigenetics is concerned with heritable changes in gene expression that occur without changes in the DNA sequence. Epigenetic changes such as DNA methylation, histone modifications, and non-coding RNAs play a central role in regulating gene activity in various biological processes, including cancer development.

In gastrointestinal cancer, epigenetic alterations have been extensively studied. In colorectal cancer, for example, researchers have identified hypermethylation of tumor suppressor genes and hypomethylation of oncogenes that contribute to tumorigenesis and progression. These techniques, in combination with the study of circulating cell-free nucleic acids, can be used for early diagnosis of colorectal neoplasms and to study genetic mechanisms of resistance to therapy [7,8,9]. For example, hypermethylation of SEPT9 is known to occur in colorectal cancer. By evaluating the methylation of SEPT9 on circulating cell-free DNA, a diagnosis can be made in the same manner as a biopsy (for this reason, it is also referred to as a liquid biopsy). A diagnostic test for colorectal cancer screening based on this technology has already been developed and shown to be useful in increasing population acceptance and effectiveness of screening compared with other methods (fecal occult blood test and colonoscopy) [10]. Similar new tests under development should further increase sensitivity and specificity in the diagnosis of precancerous lesions. From the molecular profiling of gastrointestinal tumors, researchers have identified specific immune checkpoints that tumors exploit to evade the immune system. Blocking these checkpoints with monoclonal antibodies such as pembrolizumab (anti PD-1) and ipilimumab (anti-CTLA-4) has shown remarkable efficacy in certain subsets of patients with advanced gastrointestinal cancer. In addition, advances in molecular diagnostics have enabled the identification of tumor-specific neoantigens. These neoantigens can serve as targets for personalized cancer vaccines that allow the immune system to develop a specific and robust anti-tumor response [11]. The integration of molecular biology into gastroenterology has taken the field to new heights, revealing the intricate molecular mechanisms underlying gastrointestinal health and disease. From the role of the gut microbiome in influencing overall well-being to the discovery of genetic and epigenetic factors responsible for gastrointestinal cancers, these recent discoveries have paved the way for targeted therapies and precision medicine in gastroenterology. As research continues, a deeper understanding of molecular biology will undoubtedly lead to even more breakthrough discoveries that will revolutionize the diagnosis, treatment, and management of gastrointestinal diseases, ultimately improving the quality of life for patients.
